# REV-ERBα Agonist GSK4112 attenuates Fas-induced Acute Hepatic Damage in Mice

**DOI:** 10.7150/ijms.52011

**Published:** 2021-10-25

**Authors:** Ruyue Shao, Yongqiang Yang, Kerui Fan, Xicheng Wu, Rong Jiang, Li Tang, Longjiang Li, Yi Shen, Gang Liu, Li Zhang

**Affiliations:** 1 Clinical Medical School, Chongqing Medical and Pharmaceutical College, Chongqing 401331, China.; 2 Chongqing Engineering Research Center of Pharmaceutical Sciences, Chongqing 401331, China.; 3 Department of Pathophysiology, Basic Medical College, Chongqing Medical University, Chongqing 400016, China.; 4 Laboratory of Stem Cell and Tissue Engineering, Chongqing Medical University, Chongqing 400016, China.; 5 Department of Emergency, University-Town Hospital of Chongqing Medical University, Chongqing 401331, China.

**Keywords:** Acute hepatic injury, Apoptosis, Fas, REV-ERBα, Circadian clock

## Abstract

Fas-induced apoptosis is a central mechanism of hepatocyte damage during acute and chronic hepatic disorders. Increasing evidence suggests that circadian clock plays critical roles in the regulation of cell fates. In the present study, the potential significance of REV-ERBα, a core ingredient of circadian clock, in Fas-induced acute liver injury has been investigated. The anti-Fas antibody Jo2 was injected intraperitoneally in mice to induce acute liver injury and the REV-ERBα agonist GSK4112 was administered. The results indicated that treatment of GSK4112 decreased the level of plasma ALT and AST, attenuated the liver histological changes, and promoted the survival rate in Jo2-insulted mice. Treatment with GSK4112 also downregulated the activities of caspase-3 and caspase-8, suppressed hepatocyte apoptosis. In addition, treatment with GSK4112 decreased the level of Fas and enhanced the phosphorylation of Akt. In conclusion, treatment with GSK4112 alleviated Fas-induced apoptotic liver damage in mice, suggesting that REV-ERBα agonist might have potential value in pharmacological intervention of Fas-associated liver injury.

## Introduction

Fas, a death receptor also known as CD95, is constitutively and abundantly expressed in liver, which signals the extrinsic apoptotic pathway and induces excessive hepatocyte apoptosis 1, 2. The abnormally activated Fas-induced apoptosis has been identified as a central mechanism of hepatocyte damage during acute and chronic hepatic disorders such as virus hepatitis, alcoholic liver disease, nonalcoholic fatty liver disease and ischemia/reperfusion-induced liver injury 3-5. Fas-induced liver damage can be well mimicked in experimental animal studies via injection of anti-Fas antibody (Jo2) in mice, which induces severe hepatocyte apoptosis and fulminant liver injury 6, 7. Fas-induced acute hepatic injury in mice has been widely used to investigate the pathogenesis and pharmacological targets of hepatic disorders in experimental studies 7-9.

The circadian clock is essential for the maintenance of homeostasis during a diverse of physiological processes, such as sleep-wake cycle, feeding-fasting rhythm, rhythmic change of body temperature, levels of hormones, levels of glucose and lipid, et al 10, 11. Recently, the regulatory function of circadian clock on cellular fate has been concerned 12, 13. REV-ERBα is a core ingredient of circadian clock and acts as a repressor in maintaining the circadian clock 14. Some studies have found that activation of REV-ERBα promoted apoptosis in gastric cancer cells and in palmitate-induced preadipocytes 15, 16. On the contrary, deletion of the REV-ERBα gene also increase apoptosis of neurons in internal granule cell layer during postnatal cerebellar development 17. Therefore, REV-ERBα might be a profound regulator of apoptosis, but whether it also regulate Fas-induced apoptosis remains unknown.

In the present study, to investigate the potential roles of REV-ERBα in Fas-induced acute liver damage, REV-ERBα was activated by using the REV-ERBα agonist GSK4112. GSK4112 is a selective REV-ERBα agonist that has been widely used to explore the roles of REV-ERBα in the regulation of circadian clock-associated physiological and pathophysiological events 18-21. In the present study, the potential effects of GSK4112 on the degree of hepatic damage, hepatocyte apoptosis, and the underlying mechanism were investigated.

## Materials and methods

### Reagents

REV-ERBα agonist GSK4112 was obtained from Cayman Chemical (Ann Arbor, MI, USA). Anti-Fas antibody Jo2 were supplied by BD Bioscience (Franklin Lakes, NJ, USA). The aspartate aminotransferase (AST) and alanine aminotransferase (ALT) activity detection kits were sourced from Nanjing Jiancheng Bioengineering Institute (Nanjing, China). The caspase-3 and caspase-8 activity detection kits and the Fas antibody were obtained from Beyotime Institute of Biotechnology (Jiangsu, China). The antibodies, such as β-actin, cleaved caspase-3, Akt, and p-Akt, were obtained from Cell Signaling Technology (Danvers, MA, USA). The *In situ* Cell Death Detection Kit was sourced from Roche (Indianapolis, IN, USA).

### Animals

Male C57BL/6 mice, weighing 18-22 g and 6-8 wk old, were procured from Chongqing Medical University. Throughout the experiment, the mice were maintained at specific room (20-25 °C, 12 h dark/12 h light rhythm and 45-55% relative humidity), and were provided with water and diet freely. All handling procedures about animal were authorized by the Ethics Committee of Chongqing Medical University.

### Experimental model

To establish Fas-induced acute hepatic damage, BALB/c mice were treated with Jo2 (0.5 μg/g, dissolved in normal saline (NS)) by intraperitoneal (i.p.) injection. To evaluate the effect of GSK4112 in Fas-induced hepatic damage, 32 mice were randomly allocated into four different groups (8 mice/group). In the Fas group, the mice were treated with Jo2 to establish Fas-induced hepatic damage. In the GSK4112+Fas group, GSK4112 (25 mg/kg, dissolved in DMSO) was injected intraperitoneally at 0.5 h before Jo2 exposure. The selected dosage of GSK4112 was depended on the preliminary experiments. The control group and the GSK4112 group were administered with the uniform dose of solvent or GSK4112 respectively. The animals were executed at 6 h after the treatment of Jo2 or NS. The blood was obtained for the detection of plasma index, such as ALT and AST. The livers were collected for the evaluation of morphological change, and other analysis. For assessing the role of GSK4112 on mortality, 40 mice were randomly divided into two different groups (20 mice/group), the Fas group and the GSK4112+Fas group. The mice were observed every 6 h for 7 day following Jo2 administration, and the survival rate was analyzed.

### Histological evaluation

To observe the histological change of hepatic damage, the obtained liver was fixed with buffered paraformaldehyde. The fixed specimens were embedded with paraffin. Then the paraffin waxes containing liver tissue were sliced into sections (4 μm). We stained the sections with hematoxylin and eosin (H&E), and then observed it in light microscopy. The histological abnormalities of the liver were blindly scored according to the method as previous described with slight modifications 22. Briefly, the histological changes were graded on a scale of 0-3 (0, normal; 1, mild; 2, moderate; 3, severe).

### Aminotransferase Analysis

To assess the degree of hepatic damage, the reagent kits were used to measure the levels of ALT and AST in plasma according to the program afforded by manufacturer (Nanjing Jiancheng Bioengineering Institute).

### Caspase activity assay

To detect the caspase activity, liver homogenates were prepared. The reagent kits were used to measure the activity of caspase-8 and caspase-3 according to the program afforded by manufacturer (Beyotime Institute of Biotechnology).

### TUNEL assay

To analyze the level of apoptosis in liver sections, Terminal deoxynucleotidyl transferase dUTP nick end labeling (TUNEL) was carried out with *In situ* Cell Death Detection Kit (Roche), according to the program afforded by manufacturer.

### Western blot analysis

The protein level of cleaved caspase-3, Fas, Akt and p-Akt were analyzed by Western blot. In brief, the liver tissues were lysed, and the tissue proteins were extracted by the kits of protein extraction according to the program afforded by manufacturer (Beyotime Institute of Biotechnology). The concentration of extracted protein was determined by the BCA protein assay kit (Pierce Biotechnology). Following this, the proteins with equal amount from each tissue were separated in a polyacrylamide-sodium dodecyl sulfate gel, after that, the proteins were transferred onto a nitrocellulose membrane. The membranes were incubated respectively with primary antibodies, such as cleaved caspase-3, Fas, Akt, p-Akt and β-actin, overnight at 4 °C. The next day, the membranes were incubated with the second antibody. Finally, the target blots were visualized by the ECL chemiluminescence system.

### Statistical analysis

The experimental data were expressed as mean ± standard deviation (SD). The statistical difference of groups was evaluated using one-way ANOVA with the Tukey's post hoc test. The difference of survival rate was evaluated by the Kaplan-Meier curve and log-rank test. The analysis result, *P* < 0.05, was considered to be statistical significance.

## Results

### GSK4112 attenuated Fas-induced hepatic damage

**Figure 1:**
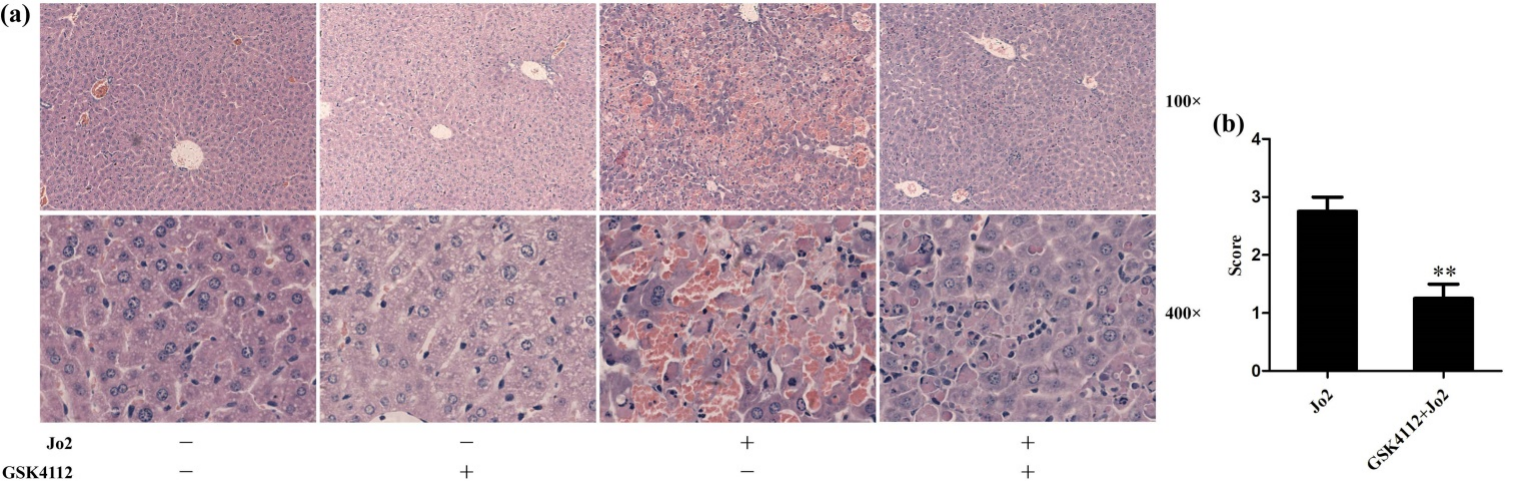
** GSK4112 attenuated Fas-induced hepatic histological damage.** The livers were collected at 6 h after Jo2 exposure. The liver tissues were embedded with paraffin and sliced into sections. The sections were stained with hematoxylin and eosin, and then observed in light microscopy. **(a)** The typical sections of each group are displayed. **(b)** The histological abnormalities of the liver were semi-quantified. **P < 0.01 compared with the Fas group (Jo2 +/GSK4112 -). n = 4.

**Figure 2:**
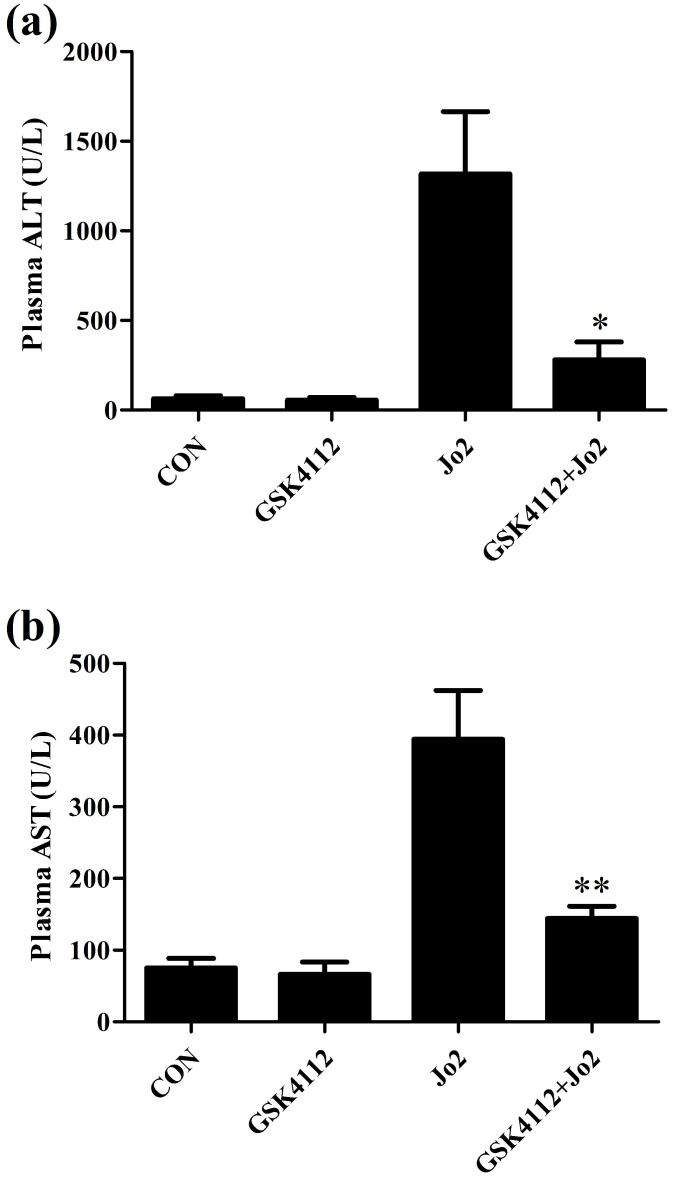
** GSK4112 decreased plasma ALT and AST induced by Jo2 exposure.** The level of plasma alanine aminotransferase (ALT) **(a)** and aspartate aminotransferase (AST) **(b)** were evaluated at 6 h after Jo2 exposure. n = 8. CON: control. *P < 0.05, **P < 0.01 compared with the Fas group (Jo2 +/GSK4112 -).

**Figure 3:**
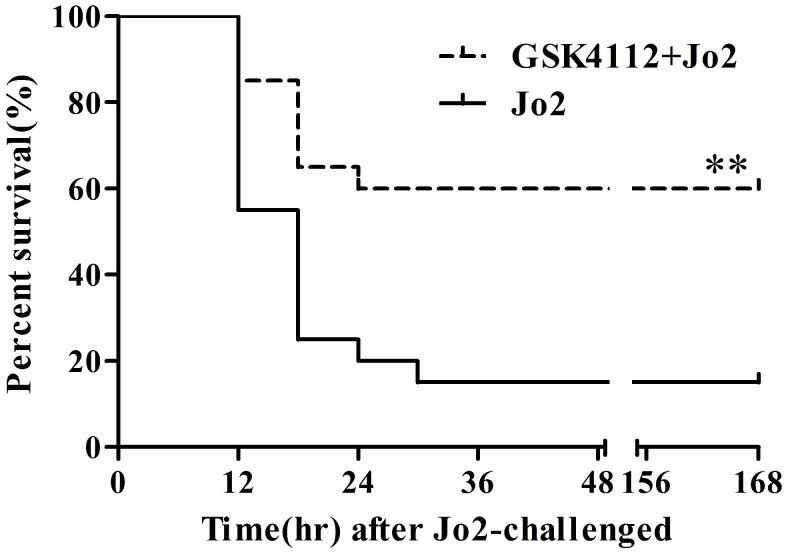
** GSK4112 reduced mortality induced by Jo2 exposure.** The survival of the experimental animals was observed every 6 h for 7 day following Jo2 administration, and the survival rate was assessed by the Kaplan-Meier curve and log-rank test. n = 20. **P <0.01 compared with the Fas group (Jo2 +/GSK4112 -).

According to the histological examination in liver sections, the control group and the GSK4112 group showed normal histological structure, but anti-Fas antibody Jo2 induced serious liver damage, including destruction of hepatic lobule, hepatocyte necrosis and hemorrhage (Fig. 1). While in the GSK4112+Fas group, the degree of liver damage was obviously ameliorated (Fig. 1). The plasma ALT and AST are considered as the representative biochemical indicators of hepatic lesion 23. Consistent with the histological abnormalities, the plasma ALT and AST increased significantly following Jo2 exposure, whereas treatment with GSK4112 suppressed the increase of ALT and AST in Jo2-exposed mice (Fig. 2). In addition, treatment with GSK4112 markedly improved the survival rate of Jo2-exposed mice (Fig. 3).

### GSK4112 suppressed Fas-induced hepatocyte apoptosis

**Figure 4:**
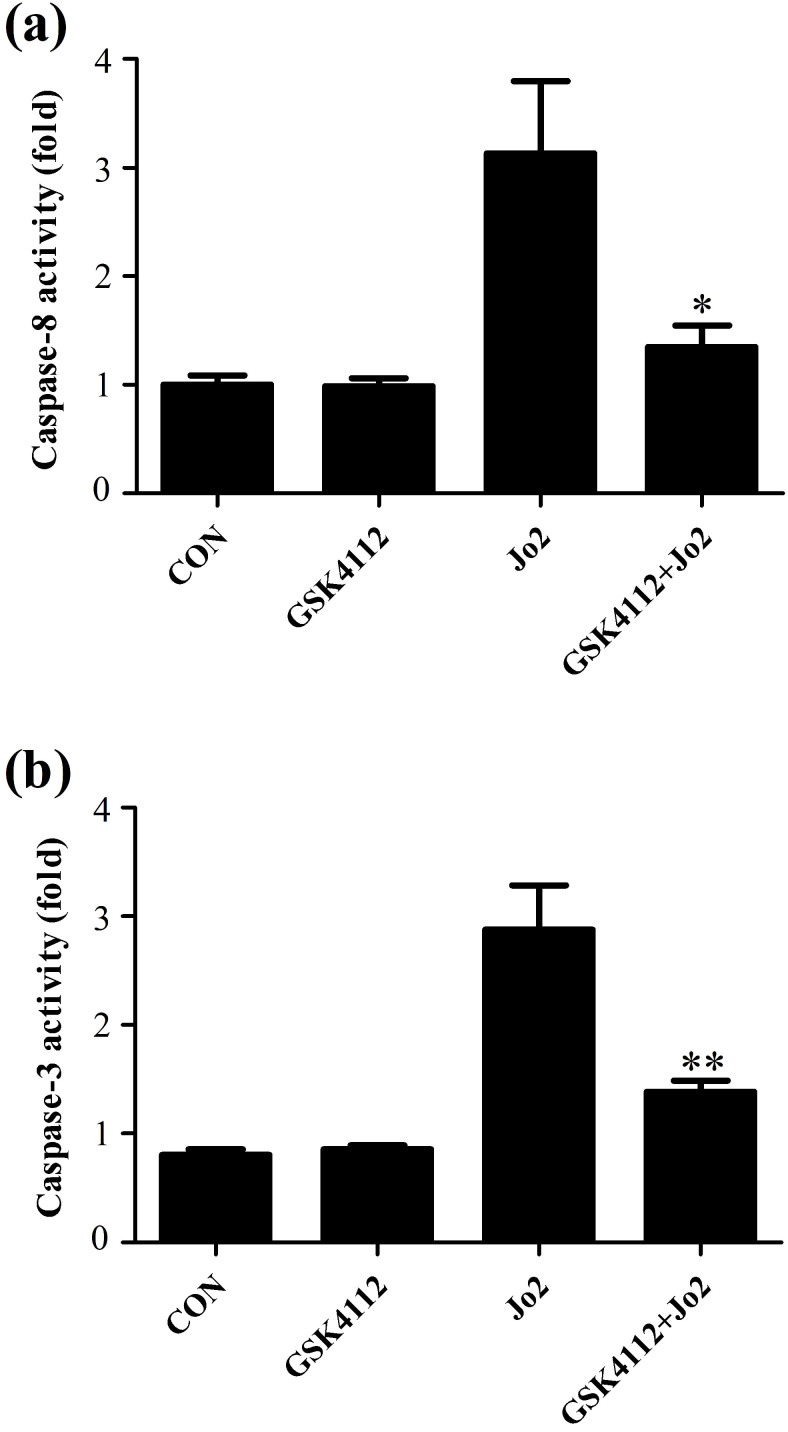
** GSK4112 decreased caspase activity after Jo2 exposure.** The livers were collected at 6 h after Jo2 exposure. The activity of caspase-8 **(a)** and caspase-3 **(b)** in the liver were evaluated. n = 8. CON: control. *P < 0.05, **P < 0.01 compared with the Fas group (Jo2 +/GSK4112 -).

**Figure 5:**
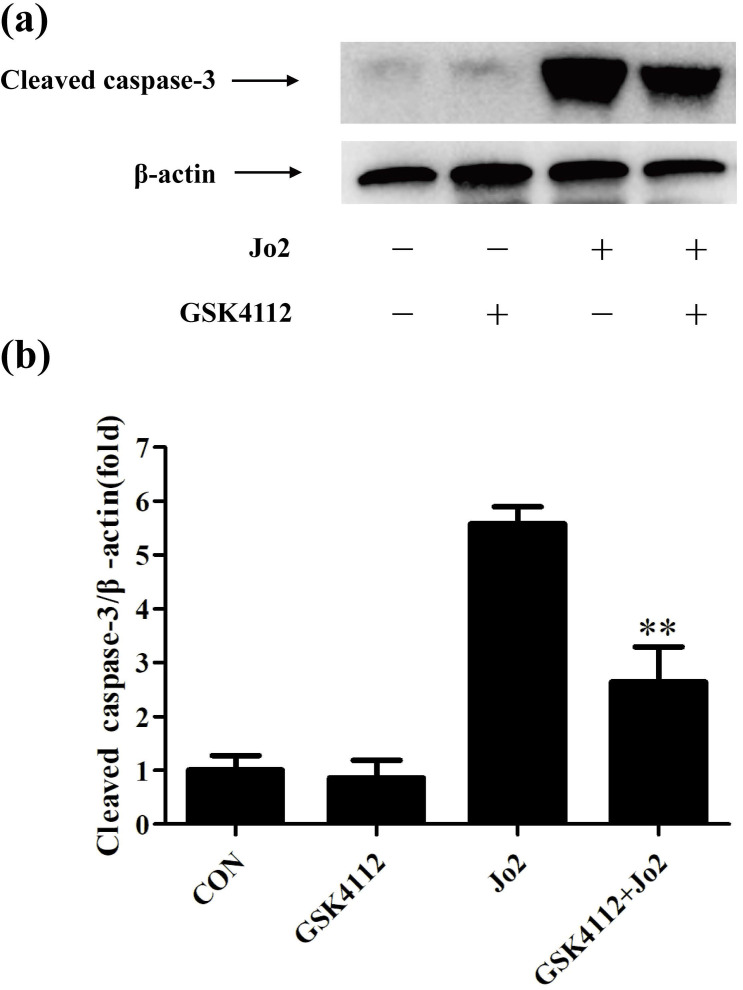
** GSK4112 suppressed the level of cleaved caspase-3 induced by Jo2 exposure.** The livers were collected at 6 h after Jo2 exposure. The protein level of cleaved caspase-3 in liver was assessed by Western blot. n = 4. CON: control. **P < 0.01 compared with the Fas group (Jo2 +/GSK4112 -).

**Figure 6:**
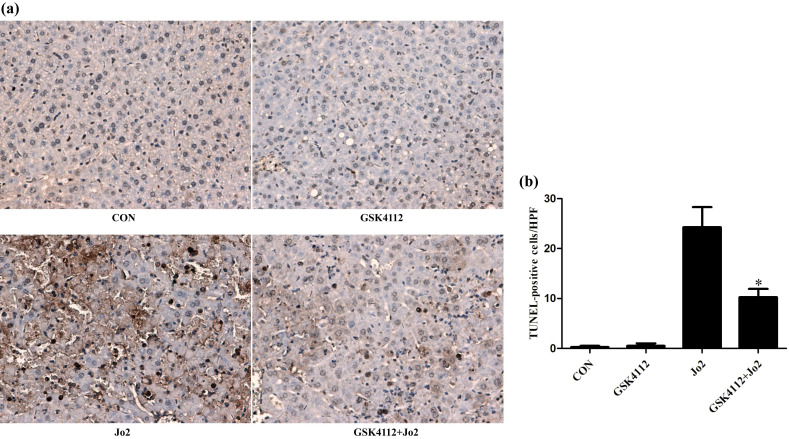
** GSK4112 attenuated hepatocyte apoptosis after Jo2 exposure.** The livers were collected at 6 h after Jo2 exposure. The sections of liver tissue were prepared and detected by TUNEL assay. The dark-brown nucleus indicates apoptotic cells. **(a)** The typical sections of each group are displayed (original magnification ×200). **(b)** The numbers of TUNEL-positive cells in 10 randomly selected field were counted under a microscope. *P < 0.05 compared with the Fas group (Jo2 +/GSK4112 -). n = 4. CON: control.

Caspase-8 is regarded as an initiator caspase, which can activate downstream caspases and trigger apoptosis 24. As expected, the challenge with Jo2 increased the activity of caspase-8 in liver tissue, but treatment with GSK4112 significantly suppressed caspase-8 activity in Jo2-exposed liver (Fig. 4a). Caspase-3 is considered as a major executor caspase in apoptosis 24. In this study, the activity of caspase-3 in liver elevated obviously after Jo2 exposure, but treatment with GSK4112 markedly suppressed the caspase-3 activity in Jo2-exposed mice (Fig. 4b). Consistently, the protein level of cleaved caspase-3 in liver increased notably after Jo2 exposure, which was reversed by the treatment with GSK4112 (Fig. 5). Accompanied with the activation of caspase-3, the TUNEL analysis showed that hepatocyte apoptosis also elevated markedly following Jo2 exposure, but this alteration was reversed by GSK4112 (Fig. 6).

### GSK4112 down-regulated the level of Fas in Jo2-exposed liver

**Figure 7:**
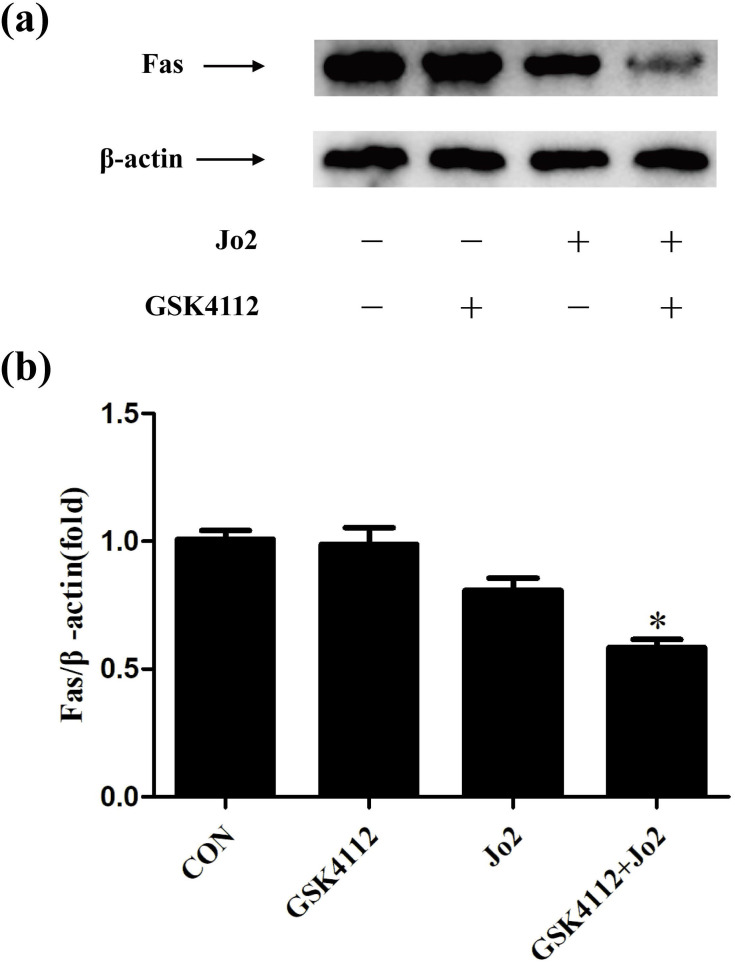
** GSK4112 down-regulated the level of Fas in Jo2-exposed liver.** The livers were collected at 6 h after Jo2 exposure. The protein level of Fas in liver was detected by Western blot, and the relative level of Fas was assessed. n = 4. CON: control. *P < 0.05 compared with the Fas group (Jo2 +/GSK4112 -).

In Fas-induced apoptosis, ligation of Fas induces the activation of caspase-8 and finally triggers cell apoptosis 2. Consistent with the suppressed activation of casepase-8 and the reduced TUNEL-positive cells, the present study found that treatment with GSK4112 down-regulated the level of Fas in Jo2-induced liver injury (Fig. 7).

### GSK4112 enhanced Akt activity in Jo2-challenged liver

**Figure 8:**
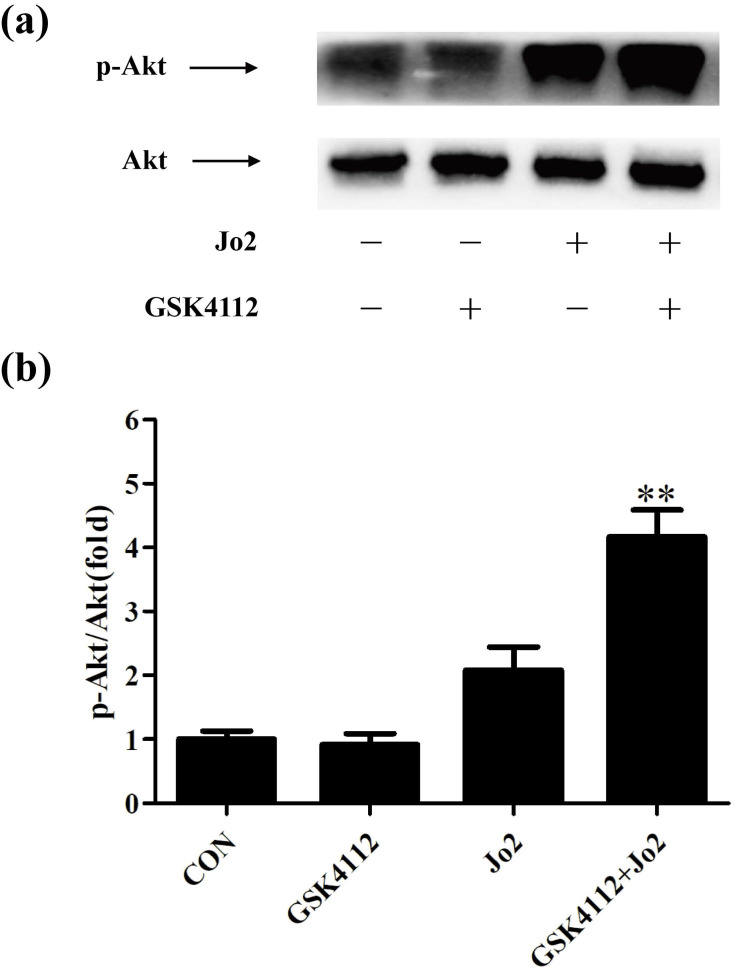
** GSK4112 enhanced Akt activity in Jo2-exposed liver.** The livers were collected at 6 h after Jo2 exposure. The protein level of Akt and p-Akt in liver was assessed by Western blot, and the relative level of p-Akt/total Akt was assessed. n = 4. CON: control. **P < 0.01 compared with the Fas group (Jo2 +/GSK4112 -).

It is well known that Akt plays a crucial role in suppressing apoptosis. Akt functions as a crucial regulator that prevents Fas-induced activation of caspase-8 25. As shown in Fig. 8, treatment with GSK4112 significantly upregulated the level of p-Akt in Jo2-exposed liver.

## Discussion

Recently, the effects of circadian clock in pathophysiologic processes have been highly concerned 12, 26. It has been found that some elements of circadian clock can regulate inflammatory response and apoptosis 27-30, but the effects of REV-ERBα in acute hepatic injury and hepatocyte apoptosis remain unclear. The present study found that treatment with REV-ERBα agonist GSK4112 provided beneficial effects in mice with Fas-induced liver damage, because GSK4112 suppressed the levels of plasma ALT and AST, attenuated the liver histological changes, and promoted the survival rate in Fas-induced mice model. Based on the above results, REV-ERBα might play a protective role in Fas-induced liver injury.

Activation of Fas induces severe hepatocyte apoptosis in mice 6, 7. In the process of apoptosis, caspase-3 is the crucial downstream executor 24. Once caspase-3 is cleaved and activated, it cleaves a mass of cellular substrates, and leads to cell apoptosis 24. In the principle of Fas-induced apoptosis, ligation of Fas results in the formation of death-inducing signaling complex (DISC) 2, 31. The DISC induces the autocatalytic processing and activation of caspase-8 2, 31. Subsequently, the activated caspase-8 cleaves and activates the effector caspases-3, and finally triggers cell apoptosis 2, 31. In the present study, treatment with GSK4112 suppressed the activity of caspase-8, caspase-3 and the level of cleaved caspase-3 in Fas-challenged mice. Consistent with the results, TUNEL assay also shown that GSK4112 decreased hepatocyte apoptosis in Fas-induced liver injury. Hence, the protective role of REV-ERBα in liver injury maybe attribute to its suppressive effects on hepatocyte apoptosis.

In agree with the results in the present study, treatment of REV-ERBα agonist SR9009 decreased the level of cleaved caspase-3 and the number of TUNEL-positive cells in the hippocampus in mice with the pilocarpine-induced status epilepticus 32. In addition, treatment of GSK4112 inhibited the activation of caspase-3 in SH-SH5Y cells 33. Previous studies have shown that mice lacking REV-ERBα gene enhanced apoptosis of neurons in the internal granule cell layer during postnatal cerebellar development 17. These data indicate that REV-ERBα plays an important effect in suppressing apoptosis.

To investigate the mechanisms through which GSK4112 suppresses the caspase cascade and hepatocyte apoptosis, the level of Fas has been determined. Interestingly, the present study found that treatment with GSK4112 down-regulated the level of hepatic Fas in Jo2-exposed mice, which might be responsible for the suppressed activation of casepase-8, the reduced TUNEL-positive cells and the alleviated liver injury in the present study. These results revealed the novel association between REV-ERBα and Fas in liver, but the underlying mechanisms remain to be further investigated.

In addition to Fas level, the Akt pathway, which plays critical roles in cell survival and apoptosis 25, has been concerned. The activated Akt prevented DISC assembly by impairing recruitment of procaspase-8, which suppresses Fas-induced activation of caspase-8 and cellular apoptosis 34. Previous study has found that treatment with SC79, an Akt activator, prevented the recruitment of procaspase-8 into the DISC, declined the activation of caspase-8, and alleviated apoptosis of hepatocytes induced by anti-Fas antibody 35. In the present study, treatment with GSK4112 upregulated the level of p-Akt in Fas-induced liver injury. In agreement with our findings, a recent study found that treatment with the REV-ERBα agonist SR9009 regulated macrophage polarization via activation of the Akt pathway 36. It has been reported that p53 is crucial for the upregulation of Fas expression in liver 37, 38, while Akt functions as a negative regulator of p53 39, 40. Therefore, Akt-p53-Fas axis might be a potential mechanism contributing to the suppressive effects of REV-ERBα agonist GSK4112 on Fas.

It is still a question how GSK4112 enhances the activity of Akt in acute hepatic injury. Previous studies have shown that PI3K and PTEN are major upstream regulator of AKT 41, 42. When PI3K is activated by diverse growth factor, PI3K phosphorylates the phosphatidylinositol (4,5)-bisphosphate (PIP2) to generate the phosphatidylinositol (3,4,5)-trisphosphate (PIP3), and then, PIP3 activates AKT 41, 42. However, the PI3K/AKT signaling pathway is negatively regulated by PTEN, which is able to dephosphorylate PIP3 and create PIP2, and inhibits the activation of AKT 41, 42. It will be an important question whether PTEN/PI3K/Akt signaling pathway plays an important role in the protective effect of GSK4112 in acute hepatic injury.

In the present study, treatment with GSK4112 significantly improved the survival of Jo2-exposed mice, suggesting that GSK4112 as well as other REV-ERBα agonists might have potential value for the treatment with Fas-associated liver damage. However, treatment with GSK4112 could not completely prevent Jo2-induced mortality, implying that REV-ERBα partially regulate Fas-induced hepatocyte apoptosis and some REV-ERBα-independent molecular events responsible for Fas-induced apoptosis remain to be further investigated. In addition, it is possible that the pharmacological reagent used in the present study might have some off-target effects. Therefore, the roles of REV-ERBα in acute liver injury should be further investigated in REV-ERBα knock-out mice.

In conclusion, the present study found that treatment with the REV-ERBα agonist GSK4112 effectively suppressed Fas-induced hepatocyte apoptosis and liver damage in mice, and the protective effects of GSK4112 might be associated with the reduced level of Fas and the enhanced activation of Akt. Although the molecular mechanisms underlying the association between REV-ERBα and Fas remain to be further investigated, the present study suggests that REV-ERBα agonist, including GSK4112, might have therapeutic benefits in Fas-associated hepatic disorders.

## References

[1] Akazawa Y, Gores GJ (2007). Death receptor-mediated liver injury. Semin Liver Dis.

[2] Lavrik IN, Krammer PH (2012). Regulation of CD95/Fas signaling at the DISC. Cell Death Differ.

[3] Yin XM, Ding WX (2003). Death receptor activation-induced hepatocyte apoptosis and liver injury. Curr Mol Med.

[4] Alkhouri N, Alisi A, Okwu V, Matloob A, Ferrari F, Crudele A (2015). Circulating Soluble Fas and Fas Ligand Levels Are Elevated in Children with Nonalcoholic Steatohepatitis. Dig Dis Sci.

[5] Al-Saeedi M, Steinebrunner N, Kudsi H, Halama N, Mogler C, Buchler MW (2018). Neutralization of CD95 ligand protects the liver against ischemia-reperfusion injury and prevents acute liver failure. Cell Death Dis.

[6] Ogasawara J, Watanabe-Fukunaga R, Adachi M, Matsuzawa A, Kasugai T, Kitamura Y (1993). Lethal effect of the anti-Fas antibody in mice. Nature.

[7] Yin XM, Wang K, Gross A, Zhao Y, Zinkel S, Klocke B (1999). Bid-deficient mice are resistant to Fas-induced hepatocellular apoptosis. Nature.

[8] Yao L, Chen W, Han C, Wu T (2016). Microsomal prostaglandin E synthase-1 protects against Fas-induced liver injury. Am J Physiol Gastrointest Liver Physiol.

[9] Weerasinghe SV, Park MJ, Portney DA, Omary MB (2016). Mouse genetic background contributes to hepatocyte susceptibility to Fas-mediated apoptosis. Mol Biol Cell.

[10] Reinke H, Asher G (2019). Crosstalk between metabolism and circadian clocks. Nat Rev Mol Cell Biol.

[11] Sinturel F, Petrenko V, Dibner C (2020). Circadian Clocks Make Metabolism Run. J Mol Biol.

[12] Gaddameedhi S, Selby CP, Kemp MG, Ye R, Sancar A (2015). The circadian clock controls sunburn apoptosis and erythema in mouse skin. J Invest Dermatol.

[13] Li Y, Cheng S, Li L, Zhao Y, Shen W, Sun X (2019). Light-exposure at night impairs mouse ovary development via cell apoptosis and DNA damage. Biosci Rep.

[14] Ikeda R, Tsuchiya Y, Koike N, Umemura Y, Inokawa H, Ono R (2019). REV-ERBalpha and REV-ERBbeta function as key factors regulating Mammalian Circadian Output. Sci Rep.

[15] Wang X, Wang N, Wei X, Yu H, Wang Z (2018). REV-ERBalpha reduction is associated with clinicopathological features and prognosis in human gastric cancer. Oncol Lett.

[16] Chu G, Zhou X, Hu Y, Shi S, Yang G (2019). Rev-erbalpha Inhibits Proliferation and Promotes Apoptosis of Preadipocytes through the Agonist GSK4112. Int J Mol Sci.

[17] Chomez P, Neveu I, Mansen A, Kiesler E, Larsson L, Vennstrom B (2000). Increased cell death and delayed development in the cerebellum of mice lacking the rev-erbA(alpha) orphan receptor. Development.

[18] Chen H, Chu G, Zhao L, Yamauchi N, Shigeyoshi Y, Hashimoto S (2012). Rev-erbalpha regulates circadian rhythms and StAR expression in rat granulosa cells as identified by the agonist GSK4112. Biochem Biophys Res Commun.

[19] Kojetin DJ, Burris TP (2011). A role for rev-erbalpha ligands in regulation of adipogenesis. Curr Pharm Des.

[20] Sundar IK, Rashid K, Sellix MT, Rahman I (2017). The nuclear receptor and clock gene REV-ERBalpha regulates cigarette smoke-induced lung inflammation. Biochem Biophys Res Commun.

[21] Chandra V, Bhagyaraj E, Nanduri R, Ahuja N, Gupta P (2015). NR1D1 ameliorates Mycobacterium tuberculosis clearance through regulation of autophagy. Autophagy.

[22] Lv H, Qi Z, Wang S, Feng H, Deng X, Ci X (2017). Asiatic Acid Exhibits Anti-inflammatory and Antioxidant Activities against Lipopolysaccharide and d-Galactosamine-Induced Fulminant Hepatic Failure. Front Immunol.

[23] Ozer J, Ratner M, Shaw M, Bailey W, Schomaker S (2008). The current state of serum biomarkers of hepatotoxicity. Toxicology.

[24] Kumar S (2007). Caspase function in programmed cell death. Cell Death Differ.

[25] Liu W, Jing ZT, Xue CR, Wu SX, Chen WN, Lin XJ (2019). PI3K/AKT inhibitors aggravate death receptor-mediated hepatocyte apoptosis and liver injury. Toxicol Appl Pharmacol.

[26] Yang Y, Yuan G, Xie H, Wei T, Zhu D, Cui J (2019). Circadian clock associates with tumor microenvironment in thoracic cancers. Aging (Albany NY).

[27] Griffin P, Dimitry JM, Sheehan PW, Lananna BV, Guo C, Robinette ML (2019). Circadian clock protein Rev-erbalpha regulates neuroinflammation. Proc Natl Acad Sci U S A.

[28] Qin T, Lu XT, Li YG, Liu Y, Yan W, Li N (2018). Effect of Period 2 on the proliferation, apoptosis and migration of osteosarcoma cells, and the corresponding mechanisms. Oncol Lett.

[29] Ma Z, Jin X, Qian Z, Li F, Xu M, Zhang Y (2019). Deletion of clock gene Bmal1 impaired the chondrocyte function due to disruption of the HIF1alpha-VEGF signaling pathway. Cell Cycle.

[30] Dakup PP, Porter KI, Gajula RP, Goel PN, Cheng Z, Gaddameedhi S (2020). The circadian clock protects against ionizing radiation-induced cardiotoxicity. FASEB J.

[31] Brint E, O'Callaghan G, Houston A (2013). Life in the Fas lane: differential outcomes of Fas signaling. Cell Mol Life Sci.

[32] Yue J, He J, Wei Y, Shen K, Wu K, Yang X (2020). Decreased expression of Rev-Erbalpha in the epileptic foci of temporal lobe epilepsy and activation of Rev-Erbalpha have anti-inflammatory and neuroprotective effects in the pilocarpine model. J Neuroinflammation.

[33] Guo DK, Zhu Y, Sun HY, Xu XY, Zhang S, Hao ZB (2019). Pharmacological activation of REV-ERBalpha represses LPS-induced microglial activation through the NF-kappaB pathway. Acta Pharmacol Sin.

[34] Jones RG, Elford AR, Parsons MJ, Wu L, Krawczyk CM, Yeh WC (2002). CD28-dependent activation of protein kinase B/Akt blocks Fas-mediated apoptosis by preventing death-inducing signaling complex assembly. J Exp Med.

[35] Liu W, Jing ZT, Wu SX, He Y, Lin YT, Chen WN (2018). A Novel AKT Activator, SC79, Prevents Acute Hepatic Failure Induced by Fas-Mediated Apoptosis of Hepatocytes. Am J Pathol.

[36] Cui L, Jin X, Xu F, Wang S, Liu L, Li X (2021). Circadian rhythm-associated Rev-erbalpha modulates polarization of decidual macrophage via the PI3K/Akt signaling pathway. Am J Reprod Immunol.

[37] Muller M, Wilder S, Bannasch D, Israeli D, Lehlbach K, Li-Weber M (1998). p53 activates the CD95 (APO-1/Fas) gene in response to DNA damage by anticancer drugs. J Exp Med.

[38] Lin P, Bush JA, Cheung KJ Jr, Li G (2002). Tissue-specific regulation of Fas/APO-1/CD95 expression by p53. Int J Oncol.

[39] Yamaguchi A, Tamatani M, Matsuzaki H, Namikawa K, Kiyama H, Vitek MP (2001). Akt activation protects hippocampal neurons from apoptosis by inhibiting transcriptional activity of p53. J Biol Chem.

[40] Ogawara Y, Kishishita S, Obata T, Isazawa Y, Suzuki T, Tanaka K (2002). Akt enhances Mdm2-mediated ubiquitination and degradation of p53. J Biol Chem.

[41] Zhang M, Zhang X (2019). The role of PI3K/AKT/FOXO signaling in psoriasis. Arch Dermatol Res.

[42] Naderali E, Khaki AA, Rad JS, Ali-Hemmati A, Rahmati M, Charoudeh HN (2018). Regulation and modulation of PTEN activity. Mol Biol Rep.

